# Middle East Respiratory Syndrome Coronavirus in Dromedaries in Ethiopia Is Antigenically Different From the Middle East Isolate EMC

**DOI:** 10.3389/fmicb.2019.01326

**Published:** 2019-06-19

**Authors:** Kazuya Shirato, Simenew Keskes Melaku, Kengo Kawachi, Naganori Nao, Naoko Iwata-Yoshikawa, Miyuki Kawase, Wataru Kamitani, Shutoku Matsuyama, Tesfaye Sisay Tessema, Hiroshi Sentsui

**Affiliations:** ^1^ Department of Virology III, National Institute of Infectious Diseases, Musashimurayama, Japan; ^2^ Department of Biotechnology, Addis Ababa Science and Technology University, Addis Ababa, Ethiopia; ^3^ Laboratory of Clinical Research on Infectious Diseases, Department of Pathogen Molecular Biology, Research Institute for Microbial Diseases, Osaka University, Suita, Japan; ^4^ Department of Pathology, National Institute of Infectious Diseases, Musashimurayama, Japan; ^5^ Institute of Biotechnology, Addis Ababa University, Addis Ababa, Ethiopia; ^6^ Laboratory of Veterinary Epizootiology, Department of Veterinary Medicine, Nihon University, Fujisawa, Japan

**Keywords:** Middle East respiratory syndrome, Middle East respiratory syndrome coronavirus, Ethiopia, dromedary, neutralization, antigenicity

## Abstract

Middle East respiratory syndrome (MERS) is an emerging respiratory disease caused by the MERS coronavirus (MERS-CoV). MERS has been endemic to Saudi Arabia since 2012. The reservoir of MERS-CoV is the dromedary camel, suggesting that MERS is primarily a zoonotic disease. MERS-CoV is common in dromedaries throughout the Middle East, North Africa, and East Africa as evidenced by neutralizing antibodies against MERS-CoV; however, human cases have remained limited to the Middle East. To better understand the cause of this difference, the virological properties of African camel MERS-CoV were analyzed based on the spike (S) protein in Ethiopia. Nasal swabs were collected from 258 young dromedaries (≤ 2 years old) in the Afar region of Ethiopia, of which 39 were positive for MERS-CoV, as confirmed by genetic tests. All positive tests were exclusive to the Amibara woreda region. Using next-generation sequencing, two full-length genomes of Amibara isolates were successfully decoded; both isolates belonged to the C2 clade based on phylogenetic analysis of full-length and S protein sequences. Recombinant EMC isolates of MERS-CoV, in which the S protein is replaced with those of Amibara isolates, were then generated to test the roles of these proteins in viral properties. Amibara S recombinants replicated more slowly in cultured cells than in EMC S recombinants. In neutralizing assays, Amibara S recombinants were neutralized by lower concentrations of sera from both Ethiopian dromedaries and EMC isolate (wild-type)-immunized mouse sera, relative to the EMC S recombinants, indicating that viruses coated in the Amibara S protein were easier to neutralize than the EMC S protein. Neutralization experiments performed using S1/S2 chimeric recombinants of the EMC and Amibara S proteins showed that the neutralization profile was dependent on the S1 region of the S protein. These results suggest that the slower viral replication and the ease of neutralization seen in the Ethiopian MERS-CoV are due to strain-specific differences in the S protein and may account for the absence of human MERS-CoV cases in Ethiopia.

## Introduction

Middle East respiratory syndrome (MERS) is an emerging respiratory disease caused by the MERS coronavirus (MERS-CoV), which has been endemic to Saudi Arabia since 2012 (Danielsson et al., 2012; Zaki et al., 2012; Assiri et al., 2013; De Groot et al., 2013; Azhar et al., 2014). As of May 22, 2019, there have been 2,428 confirmed cases in 27 countries, resulting in 838 deaths [The World Health Organization (WHO), Middle East respiratory syndrome coronavirus (MERS-CoV), https://www.who.int/emergencies/mers-cov/en/]. The dromedary camel is the primary reservoir of MERS-CoV, with evidence of circulating virus in camels in Saudi Arabia since at least 1992 (Hemida et al., 2013; Kupferschmidt, 2013; Alagaili et al., 2014; Azhar et al., 2014; Haagmans et al., 2014). MERS-CoV is transmitted to humans through close contact with dromedaries, suggesting that MERS is a zoonotic disease (Azhar et al., 2014). The seropositive rate in dromedary camels is quite high in the Middle East and in Northern and Eastern African countries (Hemida et al., 2014; Muller et al., 2014; Miguel et al., 2017; Fukushi et al., 2018; Harcourt et al., 2018), with lower rates seen outside of these regions, ranging from 30 to 50% in Tunisia to only 4.1 to 13% in the Canary Islands (Reusken et al., 2014; Gutierrez et al., 2015). Furthermore, dromedaries in Australia and Japan have been shown to be free of MERS-CoV infection (Hemida et al., 2014; Shirato et al., 2015).

Despite widespread seropositivity for MERS-CoV reported in dromedaries throughout the Middle East, North Africa, and East Africa, human MERS cases have been observed only in the Middle East, with no human cases reported in Africa to date. In a test of patient exposure, Liljander et al. (2016) reported two cases of seropositivity among 1,122 specimens tested in 2013 to 2014 in Kenya. However, this seropositivity rate among patients in Kenya (0.18%) was similar to that observed in Saudi Arabia (0.15%; Muller et al., 2015), suggesting similar rates of exposure between populations. Differences in access to public health facilities between the regions have been proposed as a possible explanation for this wide disparity in MERS-CoV cases (Liljander et al., 2016), but a definitive cause remains unclear. Although underreporting may account for some differences in reporting rates, we hypothesized that virological differences between African and Middle Eastern MERS-CoV strains may play a role in human MERS-CoV infection. To test this hypothesis, we analyzed the viral properties of African MERS-CoV obtained from nasal specimens of young dromedaries (≤2 years old) in the Afar region of Ethiopia, paying special attention to the spike (S) protein.

## Materials and Methods

### Cells and Virus

Vero (CCL-81) cells were obtained from the American Tissue Culture Collection (ATCC) and cultured in Dulbecco’s modified Eagle medium (DMEM; Sigma-Aldrich, St. Louis, MO, USA) supplemented with 5% fetal calf serum (5% FCS-DMEM). Vero/TMPRSS2 cells constitutively expressing type II transmembrane serine protease (TMPRSS2; Shirogane et al., 2008), which enhances cell entry and fusion formation of MERS-CoV (Gierer et al., 2013; Shirato et al., 2013), were maintained in 5% FCS-DMEM containing 1 mg/ml of G418 (Enzo Biochem, New York, NY, USA). BHK cells, obtained from the Health Science Research Resources Bank (HSRRB; Osaka, Japan), were maintained in 10% FCS-MEM. Calu-3 cells maintained in our institute were cultured in 10% FCS-DMEM. The MERS-CoV EMC isolate was kindly provided by Ron A. M. Fouchier, Erasmus Medical Center, Rotterdam, the Netherlands. MERS-CoV was propagated and titrated using Vero/TMPRSS2 cells as described previously (Shirato et al., 2013). Viral titer was expressed as plaque forming units (PFU). All MERS-CoVs, including recombinants, were handled in a BSL-3 laboratory under the approval of the Committee on Biorisk Management at the National Institute of Infectious Diseases.

### Specimen Collection in Ethiopia

Sera were collected from dromedaries in the Afar region of Ethiopia in March and August 2013 (Fukushi et al., 2018). A total of 184 dromedaries were sampled, including 153 females and 23 males, with a median age of 6 years; the age and sex of 8 of the animals were unknown. Nasal swabs were collected from dromedaries ≤2 years of age from four locations (Awash, Amibara, Gewane, and Semera) in the Afar region in August 2017. Specimens were collected from dromedaries using Sterile Omni Swabs (GE Healthcare, Chicago, IL, USA) wetted with distilled water. After sampling, the swabs were immediately spotted onto an FTA Classic Card with a color indicator (GE Healthcare) at each local point and stored in Multi-Barrier Pouches (GE Healthcare) containing Desiccant Packets (GE Healthcare) until used for RNA extraction.

### Detection of Middle East Respiratory Syndrome Coronavirus RNA

Spots on the FTA card were punched four times using the Harris Uni-Core Punch (6 mm; GE Healthcare). The punched cards were then resuspended in AVE buffer from the QIAamp Viral RNA Mini Kit (Qiagen, Hilden, Germany), and total RNA was isolated according to the manufacturer’s instructions. MERS-CoV RNA was detected using two QProbe RT-LAMP assays targeting the nucleocapsid (N) and open reading frame (ORF) 1a (Shirato et al., 2018b) and two Corman’s real-time RT-PCR assays (upE and ORF1a; Corman et al., 2012a,b). According to the WHO case definition, detection of at least two distinct genomic targets is required for a positive diagnosis [WHO, GAR, Revised interim case definition for reporting to WHO – Middle East respiratory syndrome coronavirus (MERS-CoV), updated on July 3, 2013, http://www.who.int/csr/disease/coronavirus_infections/case_definition/en/index.html]. The regions targeted by the four primer sets included in the Corman’s assays and QProbe RT-LAMP assays were different, and their positions in EMC isolates were as follows: upE (27,458–27,502), Corman’s ORF1a (11,197–11,252), N (28,848–29,061), and LAMP ORF1a (1,572–1,753; Supplementary Figure S1). Therefore, a positive result in at least two of these four tests fulfilled the WHO criteria, and these specimens were considered positive for MERS-CoV. In this study, two QProbe RT-LAMP assays were performed as the first screening test because they can detect Ethiopian MERS-CoV RNA within 30 min, using lyophilized reagent and a battery-driven portable tube scanner (ESEQuant TS2; Qiagen) without a large real-time RT-PCR instrument. Specimens that showed positivity on both QProbe RT-LAMP assays were considered positive for MERS-CoV. After RT-LAMP screening, specimens positive for either N or ORF1a were retested by real-time RT-PCR targeting upE and ORF1a in equipped laboratory. Specimens that were positive on at least one of the Corman’s assays were also considered positive for MERS-CoV, because these specimens had positive detection of more than two different genetic targets.

For the QProbe RT-LAMP assays, all required regents were prepared in 12-well tube strips (Ina Optika, Osaka, Japan) and then lyophilized. These were supplied by Eiken Chemical Co. Ltd. (Tokyo, Japan). Two QProbe RT-LAMP primer sets were prepared in separate wells, and 10 μL extracted RNA were diluted in 40 μl Ambion Nuclease-Free Water (Thermo Fisher Scientific, Waltham, MA, USA), after which half of the mixture (25 μl) was transferred to a well containing the dried reagent. The specimens were then immediately evaluated using the ESEQuant TS2 tube scanner (Qiagen). The two Corman’s real-time RT-PCR assays (targeting upE and ORF1a) (Corman et al., 2012a,b) were performed using the QuantiTect Probe RT-PCR Kit (Qiagen) and LightCycler 96 instrument (Roche, Basel, Switzerland).

### Sequencing Analysis

The nearly complete genome of Ethiopian MERS-CoV was analyzed by RNA sequencing. Extracted viral RNA was reverse transcribed and tagged with index adaptors using the NEBNext Ultra II RNA Library Prep Kit for Illumina (New England Biolabs, Ipswich, MA, USA) according to the manufacturer’s instructions. The resulting cDNA libraries were verified by gel electrophoresis and quantified using a Quantus Fluorometer (Promega, Madison, WI, USA). Indexed libraries were then pooled and sequenced (150-bp paired-end reads) using the MiSeq (Illumina Inc., San Diego, CA, USA) and HiSeq X Ten systems (Illumina Inc.; operated by GENEWIZ, South Plainfield, NJ, USA). Sequence reads were trimmed and mapped to the EMC isolate (GenBank accession no. JX869059) reference genome using CLC Genomic Workbench 9.0.1 software (CLC bio, Aarhus, Denmark) with the default settings, except for trimming (*Q* ≥ 20 and read length ≥ 75 bp). Finally, two full-length genome sequences were obtained and deposited into GenBank (accession nos. MK564474, camel/MERS/Amibara/118/2017; MK564475, camel/MERS/Amibara/126/2017). Phylogenetic analysis was performed using MEGA software (ver. 7.0.26; Kumar et al., 2016) with the maximum likelihood method bootstrap test.

### Generation of Recombinant Middle East Respiratory Syndrome Coronavirus

All gene recombination experiments were performed under the approval of the Committee for Experiments using Recombinant DNA and Pathogens, National Institute of Infectious Diseases (No. 28–24, as of November 21, 2016) with confirmation of the Research Promotion Bureau, Ministry of Education, Culture, Sports, Science, and Technology in Japan (No. 640, as of November 4, 2016). Recombinant MERS-CoVs were generated by a bacterial artificial chromosome (BAC) clone carrying the full-length infectious genome of the EMC isolate (designated as pBAC-MERS-wt), as described previously (Terada et al., 2017; Matsuyama et al., 2018) with modifications. The S protein sequences of the Amibara isolates were synthesized in two fragments by GeneArt Gene Synthesis (Thermo Fisher Scientific) and then inserted into the pKS336 vector (Saijo et al., 2002) using the In-Fusion HD Cloning Kit (Takara Bio, Shiga, Japan). Plasmids containing the chimeric S proteins replaced between the EMC and Amibara S sequences at the first furin site (amino acid positions 748–751 in the S protein, see Supplementary Figure S2), which marks the boundary of the S1 and S2 regions, were constructed. The S protein sequence on pBAC-MERS-wt was replaced using a Red/ET recombination system Counter-Selection BAC Modification Kit (Gene Bridges, Heidelberg, Germany). BHK cells were grown in a single well of a six-well plate in 10% FCS-MEM and transfected with 3 μg of BAC plasmid with Lipofectamine 3,000 (Thermo Fisher Scientific). After transfection, Vero/TMPRSS2 cells were detached from a single well of a six-well plate using Cell Dissociation Solution Non-Enzymatic 1× (Sigma-Aldrich), resuspended in fresh medium, and added to transfected BHK cells. The co-culture was then incubated at 37°C until cell fusions formed. After fusion, the supernatants were collected and propagated once using Vero/TMPRSS2 cells and stored as a working stock at −80°C.

### Infectivity Assays

To evaluate viral growth, cells were cultured in a 96-well plate and infected with 100 PFU of each virus. After 90 min of adsorption, the cells were washed twice with PBS, and 100 μl of DMEM containing 10% Tryptose Phosphate Broth (TPB; Sigma-Aldrich) was added. At the indicated time points, the supernatants were collected and titrated using Vero/TMPRSS2 cells as described previously (Shirato et al., 2013). Briefly, Vero/TMPRSS2 cells were formed in 96-well plate, and serially diluted supernatants were inoculated onto the cells. After 90 min of adsorption, cells were washed with PBS twice and then incubated in 10%TPB-DMEM for 24 h at 37°C. After incubation, cells were fixed with 10% formalin solution (Wako Pure Chemical Industries, Ltd., Osaka, Japan) under ultraviolet light. The fixed cells were then stained with crystal violet, and the number of cell fusions was counted under a microscope. For analysis of viral entry, 10^4^ PFU of virus was applied to Vero or Vero/TMPRSS2 cells in a 96-well plate and incubated at 37°C. After virus inoculation, cellular RNA was collected once per hour using a CellAMP Direct RNA Prep Kit (Takara Bio) for 6 h. The cells were then lysed in 50 μl processing buffer, from which 30 μl lysate was mixed with 120 μl distilled water, and 5 μl aliquots of diluted RNA were used for real-time RT-PCR. The viruses were detected indirectly by measuring subgenomic mRNA7 using TaqMan Fast Virus 1-Step Master Mix (Thermo Fisher Scientific) with the following primers and probes: forward primer, 5′-CTCGTTCTCTTGCAGAACTTTG-3′; reverse primer, 5′-GTGTTGGGTAAGCCCAGTGT-3′; probe, FAM-5′-GCACCTCGTGCTGTTTCCTTTGCC-3′-BHQ. The copy number was calculated based on the standard curve generated by real-time RT-PCR using the synthesized control RNA for mRNA7 and was expressed as copies/well.

### Neutralization Assays

For the neutralization assay, sera obtained from dromedaries as described above were used along with mice immunized with an EMS isolate (wild type) (Iwata-Yoshikawa et al., 2019). The neutralization assay for MERS-CoV was performed using Vero/TMPRSS2 cells as described (Shirato et al., 2015) with minor modifications. Serially diluted sera were mixed with 50–100 PFU of targeted viruses in 10% TPB-DMEM and incubated at room temperature for 45 min. The mixture was applied to Vero/TMPRSS2 cells in a 96-well plate, incubated for 24 h at 37°C, and then fixed with 10% formalin solution (Wako Pure Chemical Industries) under ultraviolet light. The fixed cells were then stained with crystal violet, and the number of cell fusions was counted under a microscope. Non-serum samples were used as a negative control. The serum dilution that showed 80% neutralization relative to the negative control was considered the antibody titer. Specimens showing neutralization at more than 20-fold dilution relative to the negative control were considered positive for MERS-CoV infection. A dot plot graph was drawn using KaleidaGraph ver. 4.5 (Synergy Software, Mt. Penn, PA, USA).

### Statistical Analysis

The unpaired *t* test (Student, 1908; Welch, 1947) was used to assess the statistical significance of differences between groups. In all analyses, *p* < 0.05 was taken to indicate statistical significance.

## Results

### Middle East Respiratory Syndrome Coronavirus Prevalence in Afar, Ethiopia

**Figure 1:**
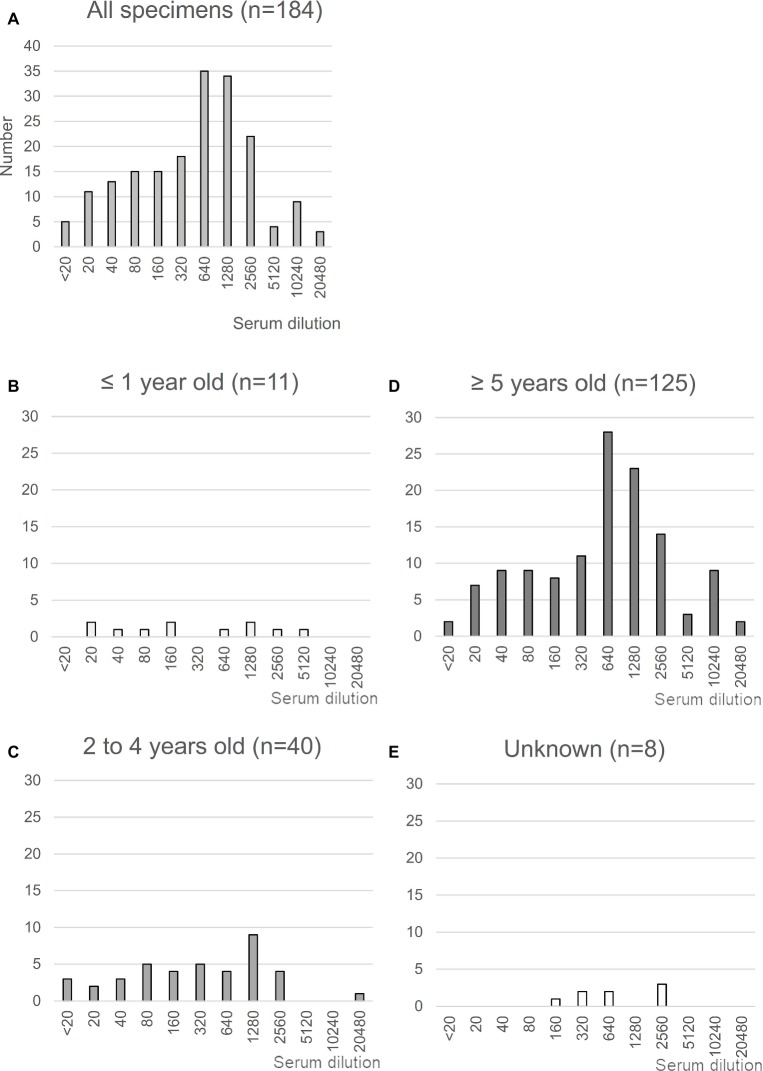
Neutralization assay using sera from Ethiopian dromedaries. **(A)** Sera were collected in the Afar region of Ethiopia in March and August 2013. The neutralization assay was performed using Vero/TMPRSS2 cells. Specimens showing reactivity at >20 dilutions were considered as positive. *n* = 184. **(B–E)** Neutralization data are shown for each age group: **(B)**, ≤1 year old (*n* = 11); **(C)**, 2–4 years old (*n* = 40); **(D)**, ≥5 years old (*n* = 125); **(E)**, age unknown (*n* = 8).

MERS-CoV is highly prevalent in North and East Africa (Hemida et al., 2014; Muller et al., 2014; Miguel et al., 2017); with >85% of dromedaries exhibiting seropositivity for MERS-CoV in Ethiopia. Using previously collected sera from dromedaries in the Afar region of Ethiopia (Fukushi et al., 2018), we first evaluated seropositivity for MERS-CoV in these animals by neutralizing assays using a wild-type EMC isolate (Figure 1). A total of 184 sera were tested, of which 179 (97.2%) were confirmed to be positive for MERS-CoV with an average titer of 430.0, consistent with the study by Miguel et al. (2017) (Figure 1A). When stratified by age, 94% (48 of 51) of dromedaries ≤4 years of age tested positive for MERS-CoV, consistent with previous results (Wernery et al., 2015), with almost half of the samples tested exhibiting antibody titers >640 (Figures 1B,C). The titers in dromedaries ≥5 years of age tended to be higher than those seen in younger animals (average titer, 247.3 vs. 519.4, respectively, *p* < 0.05; Figure 1D). Taken together, these reports strongly support the notion that MERS-CoV is endemic in Ethiopian dromedaries.

In the report of Wernery et al. (2015), only dromedaries ≤4 years of age tested positive for viral isolates or viral RNA, with the highest levels observed in animals ≤1 year old, despite higher seropositive rates in older animals. Based on these observations, we collected nasal specimens from dromedaries ≤2 years of age. In total, 258 specimens were collected, with most specimens (244) coming from animals ≤1 year old. MERS-CoV RNA was detected using genetic diagnostic methods. As shown in Table 1, 35 specimens were confirmed positive for MERS-CoV by only RT-LAMP assays (N and ORF1a). As 17 specimens were positive for a single gene in the RT-LAMP assays, they were further analyzed by real-time RT-PCR assays (upE and ORF1a), which revealed four additional positive specimens, for a final positivity rate of 15.1% (39 of 258 specimens) for MERS-CoV. These results were similar to those of the Ethiopian MERS study reported by Miguel et al. (2017), with all positive specimens detected in Amibara in the Afar region.

**Table 1:** MERS-CoV detection by RT-LAMP assays and subsequent real-time RT-PCR assays.

	Both positive	Either positive	Both negative	Total
RT-LAMP (N, ORF1a)	35	17	206	258
	Both or either positive			
RT-PCR (upE, ORF1a)		4		
		**Positive**	**Negative**	**Total**
	Total	39	219	258

### Sequence Analysis of Ethiopian Middle East Respiratory Syndrome Coronavirus

**Figure 2:**
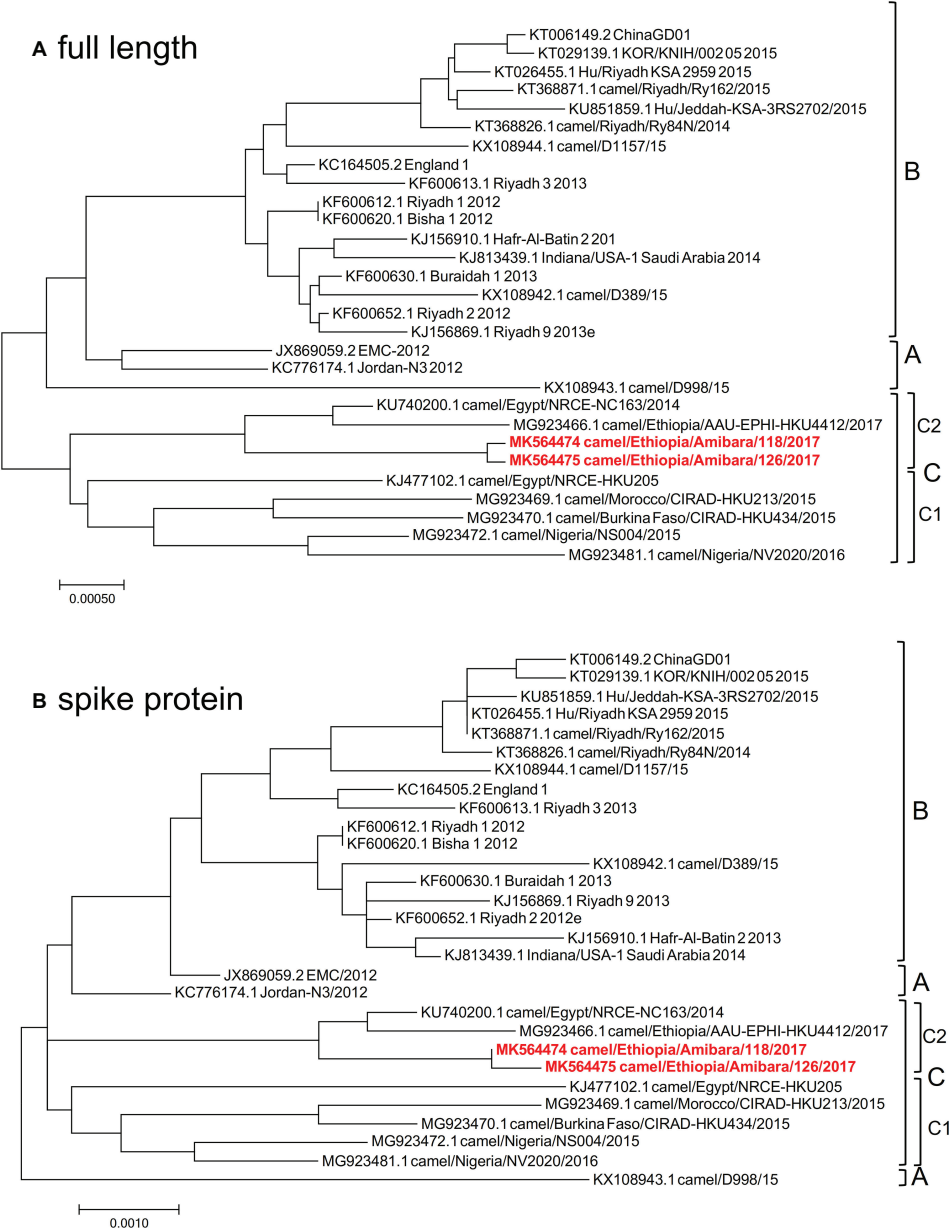
Phylogenetic analysis was performed using MEGA7 software, representing the results of **(A)** full-length and **(B)** spike protein sequences. The phylogenetic trees were constructed using the maximum likelihood method with a bootstrap test.

As Japan is one of foot and mouth disease (FMD) free countries, the import of live camel materials from Ethiopia is prohibited. Therefore, FTA Classic Cards were used in this study to fix the specimens immediately at each pastoral farm in Ethiopia. RNA extraction from the specimens was performed in Addis Ababa, Ethiopia, and then sequencing analysis was performed in Japan. Therefore, the RNAs were fragmented and could not be used for PCR amplification for next generation sequencing analysis as described by Cotten et al. (2013). To determine the genomic sequences in these specimens, RNA sequencing was performed. Among the 39 positive specimens, 12 were readily detected at early time points by RT-LAMP, suggesting high viral titers. Of these 12 specimens, nearly full-length genome sequences were detected in two specimens using MiSeq. These two specimens were then subjected to HiSeq analysis to increase the accuracy and reliability of the sequences. Finally, two independent genome sequences were obtained and designated camel/MERS/Amibara/118/2017 (MK564474) and camel/MERS/Amibara/126/2017 (MK564475). Phylogenetic analyses performed using full-length sequences showed that the Amibara isolates clustered alongside clade C isolates, with further sub-clustering into C2 alongside other Ethiopian isolates (Figure 2A). Chu et al. reported that West African MERS-CoV harbored a >360 nucleotide deletion in ORF4b relative to the EMC isolate (Chu et al., 2018). By contrast, the Amibara isolates had only two amino acids deleted from the 3′ end of ORF4b instead of the large deletion. Despite this evidence of amino acid deletions, these changes were not predicted to affect the function of ORF4b, suggesting similar activity to those in other East African MERS-CoV isolates (Kiambi et al., 2018). Furthermore, phylogenetic analysis performed using the spike (S) protein, an important protein involved in viral infection and neutralization, also showed similar clustering between isolates, suggesting that genomic attributes of East African MERS-CoV are present in the S protein (Figure 2B).

### Infectivity of Middle East Respiratory Syndrome Coronavirus Recombinants Expressing the Amibara S Protein

**Figure 3:**
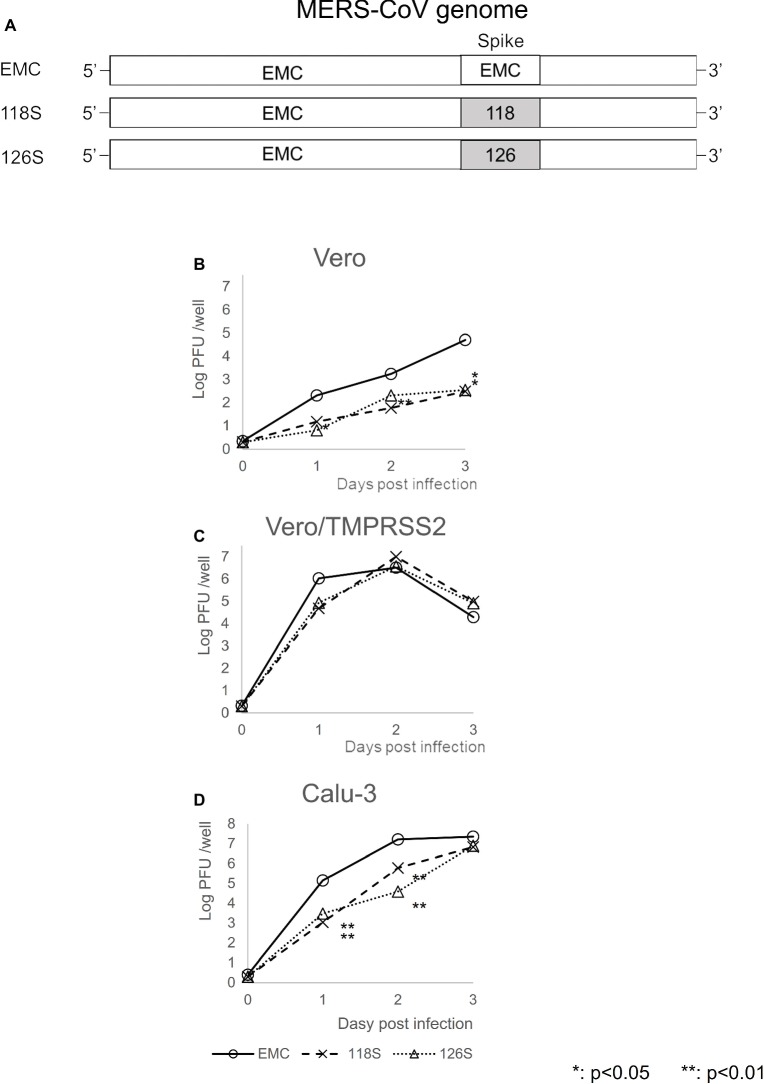
Viral replication of MERS-CoV recombinants expressing the Amibara isolate S protein. **(A)** Recombinants were recovered from BAC vector systems based on the sequence of the EMC isolate (JX869059), replacing the S protein sequence with those of Amibara S proteins. **(B–D)** Viral replication kinetics were determined using **(B)** Vero, **(C)** Vero/TMPRSS2, and **(D)** Calu-3 cells. One hundred PFU of virus were inoculated onto cells. The cells were then adsorbed, washed twice with PBS, and incubated in 10% TPB-DMEM. At 0, 1, 2, and 3 days post-inoculation, and supernatants were collected and virus titers were determined by a plaque assay using Vero/TMPRSS2 cells. Titer is expressed as PFU/well. *n* = 4; *p < 0.05; **p < 0.01.

The S protein of coronavirus shows a variety of biological functions. S1 binds to its receptor, whereas S2 is responsible for fusion activity (Sturman et al., 1985; Spaan et al., 1988; Kubo et al., 1993). The S protein also harbors most of the neutralizing antibody epitopes and is associated with pathogenicity (Spaan et al., 1988; Takase-Yoden et al., 1991; Kubo et al., 1993; Holmes and Compton, 1995). Variations in the S protein are therefore thought to directly influence important biological characteristics of the virus, such as pathogenicity and antigenicity. To evaluate the roles of these proteins in the Amibara isolates, recombinant MERS-CoVs were generated using a BAC vector system (Terada et al., 2017; Matsuyama et al., 2018) in which the S protein sequence of the EMC isolate was replaced with those of the Amibara isolates and designated as 118S and 126S (Figure 3A). The replication rates of the Amibara S recombinants were 2 logs lower than that of EMC in Vero cells until 3 days post-infection (Figure 3B). By contrast, TMPRSS2 was shown to enhance viral entry and replication of MERS-CoV (Gierer et al., 2013; Shirato et al., 2013), although the peak titers of the recombinants in Vero/TMPRSS2 cells were similar, with a slight delay in replication in the Amibara S recombinants at 1-day post-infection (Figure 3C).

Calu-3 is an immortalized human airway epithelial cell line expressing TMPRSS2 at high levels, which is commonly used for studies of respiratory pathogens due to its ability to mimic human airway tissue. Like Vero/TMPRSS2 cells, Calu-3 cells exhibited similar final viral titer peaks, although the Amibara S recombinants replicated more slowly than the EMC S recombinant (Figure 3D). These results showed that the S protein of Ethiopian MERS-CoV is associated with slower viral replication.

**Figure 4:**
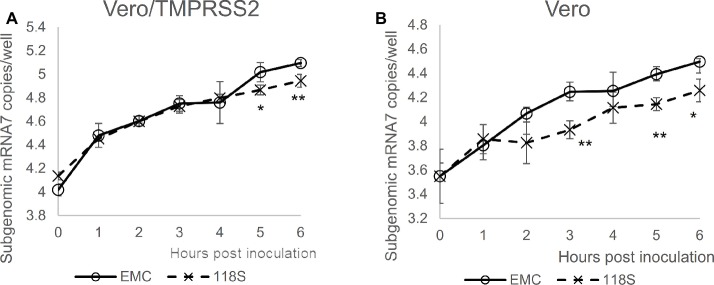
Viral entry assay. The levels of viral entry into **(A)** Vero/TMRPSS2 and **(B)** Vero cells were determined by measuring subgenomic mRNA by real-time RT-PCR assay. Ten thousand PFU of virus were inoculated onto cells in 10% TPB-DMEM, and the cells were incubated for 0 to 6 h post inoculation. Cellular RNA was then extracted using a CellAmp Direct RNA Prep Kit and used as a template for real-time RT-PCR. Copy number was calculated using standard curves drawn based on copy numbers of synthesized control RNA template, and is presented as copies/well. *n* = 4; *p < 0.05; **p < 0.01.

Next, viral entry was determined for EMC S and Amibara S (118S) recombinants by measuring subgenomic mRNA (Figure 4). In Vero/TMPRSS2 cells, the level of viral entry was identical between EMC S and 118S recombinants up to 4 h post-inoculation, after which they the level became slightly higher in the EMC S recombinant (Figure 4A). By contrast, quantification of viral entry revealed lower levels in the 118S recombinant after 2 h post-inoculation in Vero cells than the EMS S recombinant (Figure 4B). These observations suggested that the difference in viral replication may be attributed to slight differences in viral entry.

### Neutralization of Recombinant Middle East Respiratory Syndrome Coronavirus Expressing the Amibara S Protein

**Figure 5:**
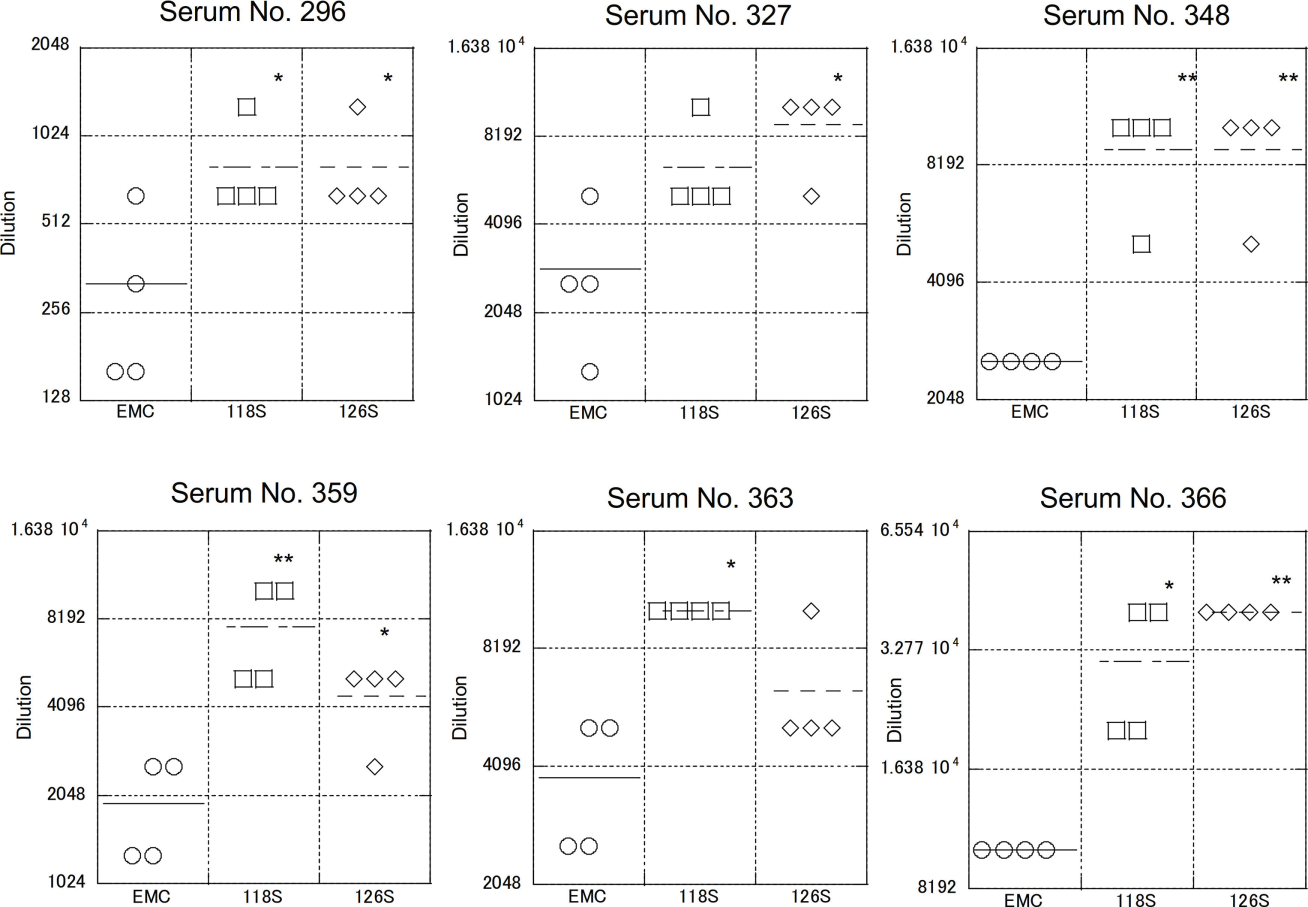
Neutralization using sera obtained from dromedaries. Sera from six dromedaries were used. Approximately 50–100 PFU of virus were mixed with serially diluted sera and incubated for 45 min at room temperature. After incubation, the mixture was inoculated onto Vero/TMPRSS2 cells followed by incubation in 10% TPB-DMEM for 1 day. The cells were then fixed with 10% formaldehyde and stained with crystal violet, and the cell fusions were counted under a microscope. Neutralization titer was defined as 80% neutralization relative to non-serum control. *n* = 4; *p < 0.05; **p < 0.01.

As described above, S protein is the primary target of neutralizing antibodies. To test strain-specific differences in antibody responses, neutralization assays examining reactivity to the Amibara S recombinants were performed using sera obtained from the Ethiopian dromedaries described in Figure 1 (Figure 5). Sera from six different animals were used (titers ranged from 320 to 10,240), with differences in reactivity calculated relative to the EMC isolate. As expected, the neutralizing titer of the EMC S recombinant was similar to that of the EMC isolate (wild type). By contrast, the neutralizing titers of Amibara S recombinants were two- to fourfold higher dilutions in all sera, meaning that the Amibara S recombinants were effectively neutralized using significantly lower antibody levels than needed for the EMC recombinant.

**Figure 6:**
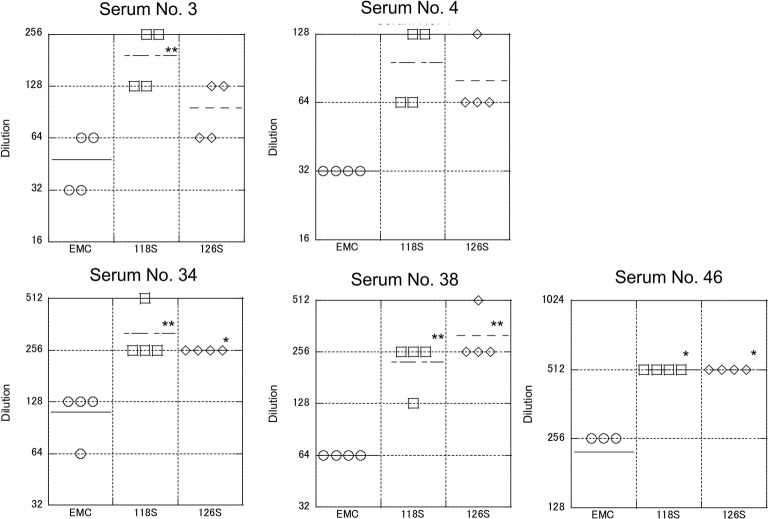
Neutralization using sera obtained from mice immunized with the EMC isolate. The mouse sera used were generated as part of a previous study (Iwata-Yoshikawa et al., 2019). Briefly, transgenic mice (C57BL/6 background) expressing human DPP4 were inoculated with the EMC isolate intranasally. After 5 or 7 days post inoculation, sera were collected under anesthesia. Five samples showing a high neutralization titer against the EMC isolate were used. The neutralization assay was performed as described in Figure 5. *n* = 4; *p < 0.05; **p < 0.01.

Although the collection dates were different, the sera used in these assays were obtained from the same area (the Afar region of Ethiopia) as the Amibara isolates. In addition, camels exported from Ethiopia to the Middle East generally do not return to Ethiopia (Faye, 2014; Eshetu and Abraham, 2016), and it is unlikely that camels carrying Middle Eastern MERS-CoV would affect the serology in Ethiopia. In addition, though yearly changes of Ethiopian MERS-CoV are unknown due to the shortage of genomic sequence database, in the neighboring country Kenya, the amino acid sequences of S1 region are identical among isolates in 2017 to 2018 (GenBank accession nos. MK357908-9 and MH734114-5, data not shown). This raises the possibility of antigenical stability in same geographical area in East Africa. Therefore, we believe that there was little divergence between sera collected in 2013 and those collected in 2017 in Ethiopia, and that the immune response to MERS-CoV in dromedaries in Afar is likely more closely related to the Amibara isolates than the EMC isolates. Thus, we suspected that there may be differences in antigenicity between the EMC and Amibara S proteins. Therefore, we performed a second neutralizing assay in mice using sera derived from mice inoculated with the EMC isolate (Iwata-Yoshikawa et al., 2019; Figure 6). Sera from five mice were used for evaluation. In contrast to our initial suspicions, the neutralization assay showed similar kinetics to those presented in Figure 4, i.e., the Amibara S recombinant strains were effectively neutralized using lower antibody concentrations than those used in the EMC S recombinant, even in EMC-immunized mouse sera. Thus, although the S protein sequence of 2017’ Amibara isolates might be different from that in 2013, when the camel sera had collected, regardless of it, the neutralizing profile among Amibara and EMC S proteins was clearly different. These results suggested that the antigenicities of EMC and Amibara S proteins are not different, but the Amibara S protein was more readily neutralized by MERS-CoV-antibodies than was the EMC S protein.

**Figure 7:**
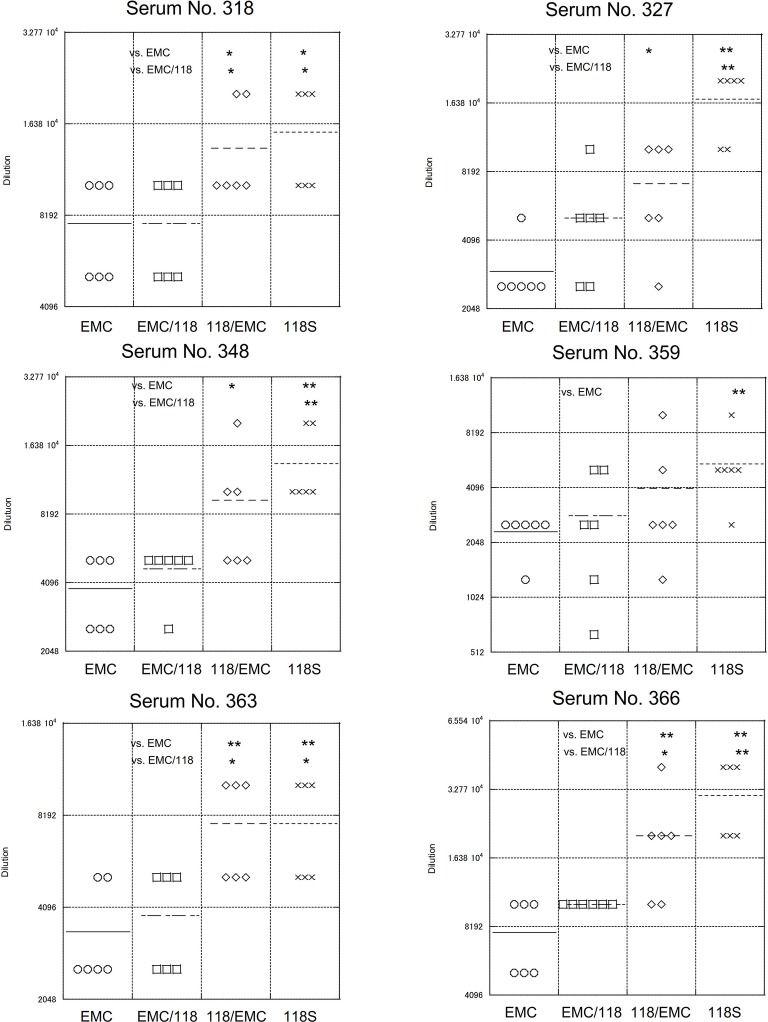
Neutralizing of recombinant MERS-CoV expressing chimeric S protein. Recombinant viruses expressing portions of the Amibara and EMC S protein, fused at the S1/S2 junction were used. Recombinant viruses were generated using a BAC vector system, as described in Figure 3. The neutralization assay was performed as described in Figure 5. *n* = 6; *p < 0.05; **p < 0.01.

Next, we assessed the contribution of the S protein to neutralization kinetics. The number of differing amino acid between the EMC and Amibara S proteins was 10 for 118S and 11 for 126S (Supplementary Figure S2), with most differences present in the S1 region. Therefore, recombinant virus strains expressing the chimeric S protein were generated by interchanging the S1 and S2 regions in EMC and Amibara S (118) proteins. Replacements were performed at the first furin site, which marks the junction between the S1 and S2 regions, yielding recombinants harboring chimeric S proteins (designated EMC/118 and 118/EMC). Neutralization assays were then performed using dromedary sera as described above (Figure 7). The results showed that the neutralizing kinetics depended primarily on the S1 region. The neutralization titers for EMC and EMC/118S were significantly lower than those for 118 EMC and 118 S. These observations indicated that the difference in neutralization profiles between the EMC and Amibara S proteins was dependent on the S1 region.

## Discussion

This study was performed to identify viral-specific differences in MERS-CoV isolates that could account for the absence of human infections in Africa, despite the similar prevalence of MERS-CoV in East and North African dromedaries. The S protein of Ethiopian MERS-CoV isolates was shown to confer slow viral replication in cells and was more easily neutralized in response to antibodies, relative to the EMC isolate. These findings suggest that differences in viral replication, and the neutralizing properties of EMC and Ethiopian MERS-CoV isolates may explain the marked discrepancy in human cases between regions. The results of this and other studies showed the Ethiopian dromedaries were highly infected with MERS-CoV (Miguel et al., 2017). The current state of human MERS-CoV infections in Ethiopia is unclear due to a lack of adequate surveillance studies, but the reported seropositive rate in humans in the neighboring country Kenya is 0.18%, providing a baseline for assessing viral exposure (Liljander et al., 2016).

The viral replication rates and ease of neutralization were different between the Amibara and EMC S recombinants, suggesting that Ethiopian MERS-CoV may be less virulent in humans due to the accumulation of these small differences. Dromedaries are commonly exported from Ethiopia to the Middle East for meat, milk production, and other purposes and are consumed at the final destination (Faye, 2014; Eshetu and Abraham, 2016). Although some exported dromedaries may harbor Ethiopian MERS-CoV variants, these Ethiopian strains may be likely to be overtaken by Middle Eastern strains similar to the EMC strain due to variations that confer lower replication rates and higher immune clearance rates. The unidirectional nature of dromedary trade and the nature of region-specific MERS-CoV variants likely may explain why human MERS-CoV cases have remained restricted to the Middle East.

One of the most important findings of this study was that the virological differences between Ethiopian and EMC isolates were based on the S protein. The coronaviral S protein is strongly associated with viral pathogenicity (Spaan et al., 1988). Following infection, the viral S protein is cleaved by host-derived proteases into two subunits: S1, which is responsible for receptor binding and neutralization, and S2, which is responsible for fusion activity (Sturman et al., 1985; Spaan et al., 1988; Kubo et al., 1993). The numbers of amino acid differences between the EMC and Amibara S proteins are 10 for 118S and 11 for 126S (Supplementary Figure S2), with most differences localized to the S1 region. The receptor binding domain (RBD) is also found in the S1 region between amino acids 367 and 606 (Chen et al., 2013; Lu et al., 2013; Mou et al., 2013; Wang et al., 2013). The results presented here show that Amibara S is more susceptible to antibody neutralization than the EMC S protein. The majority of neutralizing antibodies work by binding to the RBD, serving as a competitive inhibitor of the MERS-CoV receptor dipeptidyl peptidase 4 (DPP4; Li et al., 2015; Yu et al., 2015; Chen et al., 2017; Wang et al., 2018), or by inducing conformational changes in the S protein (Zhang et al., 2018). The amino acid differences in the RBD of EMC and Amibara S proteins are S390F, A597V, and P515L (only in 126S). Although these residues are not in direct contact with DPP4 (Wang et al., 2013; Zhang et al., 2018), substitutions in these regions may affect antibody binding affinity.

In contrast to the S1 region, the S2 region affects viral entry following cleavage by host proteases or those of co-infected bacteria (Matsuyama et al., 2005; Ami et al., 2008; Kawase et al., 2009, 2012; Shirato et al., 2013). For CoVs, the S protein is thought to be cleaved into S1 and S2 at around the first furin site (Heald-Sargent and Gallagher, 2012), although some CoVs lack furin cleavage sites. There are three amino acid differences in the S2 regions of the EMC and Amibara S proteins. The A851E substitution occurs upstream of the second furin site, the S2′ site, which is located immediately upstream of a putative fusion peptide of coronaviral spike protein (Madu et al., 2009). Cleavage at this position leads to important conformational changes (Park et al., 2016; Matsuyama et al., 2018). Therefore, this mutation may result in differences in viral entry between EMC and Amibara S recombinants. However, we reported previously that although the protease sensitivity of the human coronavirus (HCoV)-229E S protein was different between a cell-adapted laboratory strain and clinical isolates, this difference was no longer apparent after replacing two amino acids in the S2 region (Shirato et al., 2017). Similarly, differences in macrophage infectivity between feline enteric coronavirus (FECV, nonsusceptible) and feline infectious peritoneal virus (FIPV, susceptible) disappeared after introducing five amino acid substitutions (Shirato et al., 2018a). Taken together, a single mutation or a combination of mutations found in the S protein suggest clear differences associated with viral infectivity and other virulence traits. More work remains to be done to elucidate the underlying causes of S protein-derived pathogenicity in the Amibara isolate using a recombinant MERS-CoV strain.

In this study, we focused on the MERS-CoV S protein as a determinant of viral pathogenicity, although Chu et al. suggested the possible involvement of the ORF4b gene in lower replication of West African MERS-CoV (Chu et al., 2018). Although West African MERS-CoVs lacks the ORF4b gene, the absence of this gene resulted in higher type I and III interferon responses in Calu-3 cells, suggesting its potential contribution to lower viral replication (Chu et al., 2018). Differences in the public health infrastructure between Middle Eastern and African countries have often been cited as the main cause of regional deviation in MERS cases. However, in addition to the report by Chu et al. (2018), this study provided evidence of regional differences in MERS-CoVs, in which Ethiopian MERS-CoV strains exhibited lower replication rates and greater susceptibility to neutralizing antibodies than the EMC isolate. In addition, a previous genetic analysis of Middle Eastern isolates revealed an amino acid mutation at position 1,020 of the S protein, with strong selection for this variant in human MERS cases since its first appearance in 2012 (Cotten et al., 2014). This suggests a pathogenic transition of MERS-CoV during human outbreaks in the Middle East. Taken together, these observations suggest that not only the differences in public health infrastructure but also virological differences between MERS-CoV may affect regional differences in MERS cases.

Taken together, the data presented here represent the first demonstration of virological differences between Ethiopian MERS-CoV and EMC isolates driven by changes in the S protein. Further studies will be necessary to fully elucidate the pathogenicity of African MERS-CoV isolates.

## Data Availability

All datasets generated for this study are included in the manuscript and/or the Supplementary Files.

## Ethics Statement

This study used mice specimens obtained previous work (*J. Virol.* 2019. 93(6). pii: e01818–18). The specimens were collected under ethical approval as follows: Experiments using recombinant DNA and pathogens were approved by the Committee for Experiments using Recombinant DNA and Pathogens at the National Institute of Infectious Diseases, Tokyo, Japan. All animal experiments were approved by the Animal Care and Use Committee of the National Institute of Infectious Diseases and were conducted in accordance with institutional guidelines for the Care and Use of Animals. All animals were housed in a Japan Health Sciences Foundation-certified facility.

## Author Contributions

All authors participated in the planning of the project. KS, SM, TT, and HS collected specimens in Ethiopia. KS and NN performed next generation sequencing analysis. KS, KK, and WK constructed and generated MERS recombinants. KS and MK performed viral infectivity experiments. KS, MK, and NI-Y performed neutralization experiments. SM led the project. KS wrote the manuscript. All authors read and approved the final manuscript.

### Conflict of Interest Statement

The authors declare that the research was conducted in the absence of any commercial or financial relationships that could be construed as a potential conflict of interest.

## Abbreviations

BAC: Bacterial artificial chromosome
CoV: Coronavirus
DMEM: Dulbecco’s Modified Eagle’s Medium
EMC: Erasmus Medical Center
HCoV: Human coronavirus
MEM: Minimum essential medium
MERS: Middle East Respiratory Syndrome
ORF: Open reading frame
PBS: Phosphate-buffered saline
PFU: Plaque forming unit
RBD: Receptor binding domain
RT-LAMP: Reverse transcription-loop-mediated isothermal amplification
RT-PCR: Reverse transcription polymerase chain reaction
TMPRSS2: Transmembrane protease, serine 2
TPB: Tryptose phosphate broth
upE: Upstream E.
