# Supplementary material for: Middle East Respiratory Syndrome Coronavirus in Dromedaries in Ethiopia Is Antigenically Different From the Middle East Isolate EMC
Source: Front Microbiol. 2019 Jun 19;10:1326. doi: 10.3389/fmicb.2019.01326 (PMC6593072; doi:10.3389/fmicb.2019.01326)
Supplement: Supplementary file 1 [file Data_Sheet_1.PDF]

Supplemental figure 1.

For QProbe RT-LAMP

**N**	Primer sequence (5'-3', EMC, JX869059.2)	Position
N-F3	GCTCCCAGGTGGTACTTCT	28848-28866
N-B3	cagtcccctcaatgtggaag	29061-29042
N-FIP	tcatggacccaaacgatgccatACTGGAACTGGACCCGAAG	28939-28918<br>+ 28872-28890
N-BIP	GCTCCTTCAACTTTTGGGACGCtagtaccgggcgcgaatt	28956-28977<br>+ 29028-29011
N-LF	cggaatgggagtgctg	28906-28891
N-LB	GGAACCCTAACAATGATTCAGCT	28978-29000
N-QP3G（Qprobe）	GGAACCCTAACAATGATTCAGCTATTGTTACAC	28978-29010

**ORF1a**	Primer sequence (5'-3', EMC, JX869059.2)	Position
ORF1a-F3	GCCTACTTTGGATGTGAGG	1572-1590
ORF1a-B3	acaacgaactctcccaca	1753-1736
ORF1a-FIP	taaagatggagtctccaatccttgaAAGGTACTATGTACTTTGTGCC	1656-1632<br>+ 1591-1612
ORF1a-BIP	GTACTGGCTCTTGGAACAAGGagttaagggaatgctgagt	1663-1683<br>+ 1734-1716
ORF1a-LF	acaacagacttagctctag	1612-1630
ORF1a-LB	GGTCACTCAAATTGCTAACATG	1682-1703
ORF1a-QP3G（Qprobe）	GGTCACTCAAATTGCTAACATGTTCTTGGAACAGAC	1682-1717

For real-time RT-PCR

upE	Primer sequence (5'-3', EMC, JX869059.2)	Position
upE-Forward	GCAACGCGCGATTCAGTT	27458-27475
upE-Reverse	GCCTCTACACGGGACCCATA	27549-27530
upE-Probe (FAM-TAMRA)	CTCTTCACATAATCGCCCCGAGCTCG	27477-27502

upE	Primer sequence (5'-3', EMC, JX869059.2)	Position
ORF1a-Forward	CCACTACTCCCATTTCGTCAG	11197-11217
ORF1a-Reverse	CAGTATGTGTAGTGCGCATATAAGCA	11255-11280
ORF1a-Probe (FAM-TAMRA)	TTGCAAATTGGCTTGCCCCCACT	11230-11252

Supplemental figure 1. Primer and Probe sequences used for QProbe RT-LAMP and real-time RT-PCR were listed. The references were as follows: QProbe RT-LAMP, Shirato, K et al., *J Virol Methods* 258, 41-48; real-time RT-PCR, Corman, V et al., (2012). *Euro Surveill* 17, 20285 and 20334.

Supplemental figure 2.

Alignment of S protein (amino acids)

```
                  *        20         *        40         *        60         *
EMC : MIHSVFLLMFLLTPTESYVDVGPDSVKSACIEVDIQQTFFDKTWPRPIDVSKADGIIYPQGRTYSNITITYQGLFPYQ :  78
118 : MIHSVFLLMFLLTPTESYVDVGPDSAKSACIEVDIQQTFFDKTWPRPIDVSKADGIIYPQGRTYSNITITYQGLFPYQ :  78
126 : MIHSVFLLMFLLTPTESYVDVGPDSAKSACIEVDIQQTFFDKTWPRPIDVSKADGIIYPQGRTYSNITITYQGLFPYQ :  78
      MIHSVFLLMFLLTPTESYVDVGPDSaKSACIEVDIQQTFFDKTWPRPIDVSKADGIIYPQGRTYSNITITYQGLFPYQ

      80         *       100         *       120         *       140         *
EMC : GDHGDMYVYSAGHATGTTPQKLFVANYSQDVKQFANGFVVRIGAAANSTGTVIISPSTSATIRKIYPAFMLGSSVGNF : 156
118 : GDHGDMYVYSAGHATGTTPQKLFVANYSQDVKQFANGFVVRIGAAANSTGTVIISPSTSAIIRKIYPAFMLGSSVGNF : 156
126 : GDHGDMYVYSAGHATGTTPQKLFVANYSQDVKQFANGFVVRIGAAANSTGTVIISPSTSAIIRKIYPAFMLGSSVGNF : 156
      GDHGDMYVYSAGHATGTTPQKLFVANYSQDVKQFANGFVVRIGAAANSTGTVIISPSTSAiIRKIYPAFMLGSSVGNF

       160         *       180         *       200         *       220         *
EMC : SDGKMGRFFNHTLVLLPDGCGTLLRAFYCILEPRSGNHCPAGNSYTSFATYHTPATDCSDGNYNRNASLNSFKEYFNL : 234
118 : SYGKMGRFFNHTLVLLPDGCGTLLRAFYCILEPRSGNYCPAGNSYTSFATYHTPATDCSDGNYNRNASLNSFKEYFNL : 234
126 : SYGKMGRFFNHTLVLLPDGCGTLLRAFYCILEPRSGNYCPAGNSYTSFATYHTPATDCSDGNYNRNASLNSFKEYFNL : 234
      SyGKMGRFFNHTLVLLPDGCGTLLRAFYCILEPRSGNyCPAGNSYTSFATYHTPATDCSDGNYNRNASLNSFKEYFNL

         240         *       260         *       280         *       300         *
EMC : RNCTFMYTYNITEDEILEWFGITQTAQGVHLFSSRYVDLYGGNMFQFATLPVYDTIKYYSIIPHSIRSIQSDRKAWAA : 312
118 : RNCTFMYTYNITEDEILEWFGITQTAQGVHLFSSRYVDLYGGNMFQFATLPVYDTIKYYSIIPHSIRSIQSDRKAWAA : 312
126 : RNCTFMYTYNITEDEILEWFGITQTAQGVHLFSSRYVDLYGGNMFQFATLPVYDTIKYYSIIPHSIRSIQSDRKAWAA : 312
      RNCTFMYTYNITEDEILEWFGITQTAQGVHLFSSRYVDLYGGNMFQFATLPVYDTIKYYSIIPHSIRSIQSDRKAWAA

           320         *       340         *       360         *       380         *
EMC : FYVYKLQPLTFLLDFSVDGYIRRAIDCGFNDLSQLHCSYESFDVESGVYSVSSFEAKPSGSVVEQAEGVECDFSPLLS : 390
118 : FYVYKLQPLTFLLDFSVDGYIRRAIDCGFNDLSQLHCSYESFDVESGVYSVSSFEAKPSGSVVEQAEGVECDFSPLLF : 390
126 : FYVYKLQPLTFLLDFSVDGYIRRAIDCGFNDLSQLHCSYESFDVESGVYSVSSFEAKPSGSVVEQAEGVECDFSPLLF : 390
      FYVYKLQPLTFLLDFSVDGYIRRAIDCGFNDLSQLHCSYESFDVESGVYSVSSFEAKPSGSVVEQAEGVECDFSPLLf
```

```
             400         *         420         *         440         *         460
EMC : GTPPQVYNFKRLVFTNCNYNLTKLLSLFSVNDFTCSQISPAAIASNCYSSLILDYFSYPLSMKSDLSVSSAGPISQFN :  468
118 : GTPPQVYNFKRLVFTNCNYNLTKLLSLFSVNDFTCSQISPAAIASNCYSSLILDYFSYPLSMKSDLSVSSAGPISQFN :  468
126 : GTPPQVYNFKRLVFTNCNYNLTKLLSLFSVNDFTCSQISPAAIASNCYSSLILDYFSYPLSMKSDLSVSSAGPISQFN :  468
      GTPPQVYNFKRLVFTNCNYNLTKLLSLFSVNDFTCSQISPAAIASNCYSSLILDYFSYPLSMKSDLSVSSAGPISQFN

       *         480         *         500         *         520         *         540
EMC : YKQSFSNPTCLILATVPHNLTTITKPLKYSYINKCSRLLSDDRTEVPQLVNANQYSPCVSIVPSTVWEDGDYYRKQLS :  546
118 : YKQSFSNPTCLILATVPHNLTTITKPLKYSYINKCSRLLSDDRTEVPQLVNANQYSPCVSIVPSTVWEDGDYYRKQLS :  546
126 : YKQSFSNPTCLILATVPHNLTTITKPLKYSYINKCSRLLSDDRTEVLQLVNANQYSPCVSIVPSTVWEDGDYYRKQLS :  546
      YKQSFSNPTCLILATVPHNLTTITKPLKYSYINKCSRLLSDDRTEVpQLVNANQYSPCVSIVPSTVWEDGDYYRKQLS

         *         560         *         580         *         600         *         620
EMC : PLEGGGWLVASGSTVAMTEQLQMGFGITVQYGTDTNSVCPKLEFANDTKIASQLGNCVEYSLYGVSGRGVFQNCTAVG :  624
118 : PLEGGGWLVASGSTVAMTEQLQMGFGITVQYGTDTNSVCPKLEFANDTKIVSQLGNCVEYSLYGVSGRGVFQNCTAVG :  624
126 : PLEGGGWLVASGSTVAMTEQLQMGFGITVQYGTDTNSVCPKLEFANDTKIVSQLGNCVEYSLYGVSGRGVFQNCTAVG :  624
      PLEGGGWLVASGSTVAMTEQLQMGFGITVQYGTDTNSVCPKLEFANDTKIvSQLGNCVEYSLYGVSGRGVFQNCTAVG
```

Receptor binding domain

```
           *         640         *         660         *         680         *         700
EMC : VRQQRFVYDAYQNLVGYYSDDGNYYCLRACVSVPVSVIYDKETKTHATLFGSVACEHISSTMSQYSRSTRSMLKRRDS :  702
118 : VPQQRFVYDAYQNLVGYYSDDGNYYCLRACVSVPVSVIYDKETKTHATLFGSVACEHISSTMSQYSRSTRSMLKRRDS :  702
126 : VPQQRFVYDAYQNLVGYYSDDGNYYCLRACVSVPVSVIYDKETKTHATLFGSVACEHISSTMSQYSRSTRSMLKRRDS :  702
      VpQQRFVYDAYQNLVGYYSDDGNYYCLRACVSVPVSVIYDKETKTHATLFGSVACEHISSTMSQYSRSTRSMLKRRDS

             *         720         *         740         *         760         *         780
EMC : TYGPLQTPVGCVLGLVNSSLFVEDCKLPLGQSLCALPDTPSTLTPRSVRSVPGEMRLASIAFNHPIQVDQLNSSYFKL :  780
118 : TYGPLQTPVGCVLGLVNSSLFVEDCKLPLGQSLCALPDTPSTLTPRSVRSVPGEMRLASIAFNHPIQVDQLNSSYFKL :  780
126 : TYGPLQTPVGCVLGLVNSSLFVEDCKLPLGQSLCALPDTPSTLTPRSVRSVPGEMRLASIAFNHPIQVDQLNSSYFKL :  780
      TYGPLQTPVGCVLGLVNSSLFVEDCKLPLGQSLCALPDTPSTLTPRSVRSVPGEMRLASIAFNHPIQVDQLNSSYFKL
```

1st Furin site

```
                *        800         *        820         *        840         *         8
EMC : SIPTNFSFGVTQEYIQTTIQKVTVDCKQYVCNGFQKCEQLLREYGQFCSKINQALHGANLRQDDSVRNLFASVKSSQS :  858
118 : SIPTNFSFGVTQEYIQTTIQKVTVDCKQYVCNGFQKCEQLLREYGQFCSKINQALHGANLRQDDSVRNLFESVKSSQS :  858
126 : SIPTNFSFGVTQEYIQTTIQKVTVDCKQYVCNGFQKCEQLLREYGQFCSKINQALHGANLRQDDSVRNLFESVKSSQS :  858
      SIPTNFSFGVTQEYIQTTIQKVTVDCKQYVCNGFQKCEQLLREYGQFCSKINQALHGANLRQDDSVRNLFeSVKSSQS

      60         *        880         *        900         *        920         *
EMC : SPIIPGFGGDFNLTLLEPVSISTGSRSARSAIEDLLFDKVTIADPGYMQGYDDCMQQGPASARDLICAQYVAGYKVLP :  936
118 : SPIIPGFGGDFNLTLLEPVSISTGSRSARSAIEDLLFDKVTIADPGYMQGYDDCMQQGPASARDLICAQYVAGYKVLP :  936
126 : SPIIPGFGGDFNLTLLEPVSISTGSRSARSAIEDLLFDKVTIADPGYMQGYDDCMQQGPASARDLICAQYVAGYKVLP :  936
      SPIIPGFGGDFNLTLLEPVSISTGSRSARSAIEDLLFDKVTIADPGYMQGYDDCMQQGPASARDLICAQYVAGYKVLP
                              2nd Furin site
       940         *        960         *        980         *       1000         *
EMC : PLMDVNMEAAYTSSLLGSIAGVGWTAGLSSFAAIPFAQSIFYRLNGVGITQQVLSENQKLIANKFNQALGAMQTGFTT : 1014
118 : PLMDVNMEAAYTSSLLGSIAGVGWTAGLSSFAAIPFAQSIFYRLNGVGITQQVLSENQKLIANKFNQALGAMQTGFTT : 1014
126 : PLMDVNMEAAYTSSLLGSIAGVGWTAGLSSFAAIPFAQSIFYRLNGVGITQQVLSENQKLIANKFNQALGAMQTGFTT : 1014
      PLMDVNMEAAYTSSLLGSIAGVGWTAGLSSFAAIPFAQSIFYRLNGVGITQQVLSENQKLIANKFNQALGAMQTGFTT

        1020         *       1040         *       1060         *       1080         *
EMC : TNEAFQKVQDAVNNNAQALSKLASELSNTFGAISASIGDIIQRLDVLEQDAQIDRLINGRLTTLNAFVAQQLVRSESA : 1092
118 : TNEAFQKVQDAVNNNAQALSKLASELSNTFGAISASIGDIIQRLDVLEQDAQIDRLINGRLTTLNAFVAQQLVRSESA : 1092
126 : TNEAFQKVQDAVNNNAQALSKLASELSNTFGAISASIGDIIQRLDVLEQDAQIDRLINGRLTTLNAFVAQQLVRSESA : 1092
      TNEAFQKVQDAVNNNAQALSKLASELSNTFGAISASIGDIIQRLDVLEQDAQIDRLINGRLTTLNAFVAQQLVRSESA

          1100         *       1120         *       1140         *       1160         *
EMC : ALSAQLAKDKVNECVKAQSKRSGFCGQGTHIVSFVVNAPNGLYFMHVGYYPSNHIEVVSAYGLCDAANPTNCIAPVNG : 1170
118 : ALSAQLAKDKVNECVKAQSKRSGFCGQGTHIVSFVVNAPNGLYFMHVGYYPSNHIEVVSAYGLCDSANPTNCIAPVNG : 1170
126 : ALSAQLAKDKVNECVKAQSKRSGFCGQGTHIVSFVVNAPNGLYFMHVGYYPSNHIEVVSAYGLCDSANPTNCIAPVNG : 1170
      ALSAQLAKDKVNECVKAQSKRSGFCGQGTHIVSFVVNAPNGLYFMHVGYYPSNHIEVVSAYGLCDsANPTNCIAPVNG
```

```
              1180          *        1200          *        1220          *        1240
EMC : YFIKTNNTRIVDEWSYTGSSFYAPEPITSLNTKYVAPQVTYQNISTNLPPPLLGNSTGIDFQDELDEFFKNVSTSIPN : 1248
118 : YFIKTNNTRIVDEWSYTGSSFYAPEPITSLNTKYVAPQVTYQNISTNLPPPLLGNSTGIDFQDELDEFFKNVSTSIPN : 1248
126 : YFIKTNNTRIVDEWSYTGSSFYAPEPITSLNTKYVAPQVTYQNISTNLPPPLLGNSTGIDFQDELDEFFKNVSTSIPN : 1248
      YFIKTNNTRIVDEWSYTGSSFYAPEPITSLNTKYVAPQVTYQNISTNLPPPLLGNSTGIDFQDELDEFFKNVSTSIPN

       *        1260          *        1280          *        1300          *        1320
EMC : FGSLTQINTTLLDLTYEMLSLQQVVKALNESYIDLKELGNYTYYNKWPWYIWLGFIAGLVALALCVFFILCCTGCGTN : 1326
118 : FGSLTQINTTLLDLTYEMLSLQQVVKALNESYIDLKELGNYTYYNKWPWYIWLSFIAGLVALALCVFFILCCTGCGTN : 1326
126 : FGSLTQINTTLLDLTYEMLSLQQVVKALNESYIDLKELGNYTYYNKWPWYIWLSFIAGLVALALCVFFILCCTGCGTN : 1326
      FGSLTQINTTLLDLTYEMLSLQQVVKALNESYIDLKELGNYTYYNKWPWYIWLsFIAGLVALALCVFFILCCTGCGTN

         *        1340          *
EMC : CMGKLKCNRCCDRYEEYDLEPHKVHVH* : 1353
118 : CMGKLKCNRCCDRYEEYDLEPHKVHVH* : 1353
126 : CMGKLKCNRCCDRYEEYDLEPHKVHVH* : 1353
      CMGKLKCNRCCDRYEEYDLEPHKVHVH
```

Supplement figure 2. An alignment of amino acid sequences of Amibara and EMC S proteins. The alignment were constructed using GeneDoc software [Nicholas, Kb & Nicholas, Hb. (1996). GeneDoc: a tool for editing and annotating multiple sequence alignments. Distributed by the authors.]. Two furin recognition sites were enclosed by blue and green lines. The receptor binding domain was enclosed by red line referring following study: Guangwen Lu et al., (2013). Nature. 500: 227–231.
